# RL-Based Parallel LDPC Decoding with Clustered Scheduling

**DOI:** 10.3390/e28020215

**Published:** 2026-02-12

**Authors:** Yusuf Ozkan, Yauhen Yakimenka, Jörg Kliewer

**Affiliations:** Helen and John C. Hartmann Department of Electrical and Computer Engineering, New Jersey Institute of Technology, Newark, NJ 07102, USA; yo44@njit.edu (Y.O.);

**Keywords:** high-throughput decoding, message-passing scheduling, error-correcting codes, low-density parity-check codes, reinforcement learning

## Abstract

We propose a reinforcement learning (RL)-based decoding framework for high-throughput parallel decoding of low-density parity-check (LDPC) codes using clustered scheduling. Parallel LDPC decoders must balance error-correction performance and decoding latency while avoiding memory conflicts. To address this trade-off, we construct clusters of check nodes that satisfy a two-edge independence property, which enables conflict-free row-parallel belief propagation. An RL agent is trained offline to assign Q-values to clusters and to prioritize their update order during decoding. To overcome the exponential storage requirements of existing RL-based scheduling methods, we introduce the Q-Sum method, which approximates cluster-level Q-values as the sum of Q-values of individual check nodes, reducing storage complexity from exponential to linear in the number of check nodes. We further propose an On-the-Fly clustering strategy that enforces two-edge independence dynamically during decoding and provides additional flexibility when static clustering is not feasible. Simulation results for array-based LDPC codes over additive white Gaussian noise (AWGN) channels show that the proposed methods improve the latency-versus-performance trade-off of parallel LDPC decoders, achieving lower decoding latency and higher throughput while maintaining error rates comparable to state-of-the-art decoding methods.

## 1. Introduction

The Third Generation Partnership Project (3GPP) introduced a new phase called 5G-Advanced (5G-A), starting from Release 18, to address increasingly diverse and demanding use cases [1]. This development highlights new capabilities such as enhanced energy efficiency, coverage extensions, and support for artificial intelligence (AI) and machine learning (ML) techniques. The industry’s roadmap reflects a strong commitment to redefining radio access and core networks through AI and ML integration, to achieve low latency and high reliability.

The performance goals for 6G extend well beyond those of 5G, with improvements in latency, reliability, and connection density, as well as higher spectral and energy efficiency. These projected advancements highlight the higher performance demands that future LDPC decoders must meet to achieve 6G’s throughput and latency targets.

Latency and reliability are two fundamental performance metrics in modern communication networks. As next-generation systems such as 6G target 10–100× improvements in these metrics, each subsystem must meet higher performance objectives. On the receiver side, the decoder’s efficiency is crucial for achieving low-latency, high-reliability performance. In recent studies on LDPC decoding architectures [2,3,4,5,6], researchers have investigated advanced techniques from reinforcement learning-based scheduling to optimized strategies for resolving pipeline conflicts. Pipelining refers to processing different parts of the same codeword or multiple codewords simultaneously, while pipeline conflicts arise when memory access is blocked by operations from another parallel stream.

The decoding process of LDPC codes is based on iterative message-passing algorithms, such as Belief Propagation (BP) and the Min-Sum algorithm. Traditionally, a flooding schedule has been employed, where all check nodes (CNs) and variable nodes (VNs) are updated simultaneously in each iteration; however, updated messages are used only in the next iteration. Sequential (layered) decoding methods update CNs one by one, so that updated messages are available for immediate use in the same iteration and therefore provide better error-correction performance in fewer iterations [7]. However, this sequential message update requires strict data dependencies.

In contrast, pipelined decoder architectures have demonstrated that careful management of memory access and instruction flow can sustain high throughput [8].

Two main parallelization strategies have been studied in the literature. Multi-frame decoders process multiple frames in parallel, which increases throughput but requires large cache memory and raises power consumption [9]. Row-parallel layered decoding, on the other hand, processes several CNs of a single frame concurrently. This approach reduces memory conflicts and improves hardware utilization, but it also requires careful scheduling to avoid pipeline hazards [10,11]. To address these challenges, researchers have investigated dynamic scheduling and memory optimization techniques [12,13]. In addition, advances in memory fabrics and interconnect designs have reduced latency and overhead, enabling scalability to beyond 1 Gb/s decoding throughput [14].

LDPC codes are inherently scalable, supporting a wide range of code rates and block lengths. This makes them adaptable for diverse applications, from low-rate error correction in Internet-of-Things (IoT) devices to high-rate data transmission in eMBB systems. The BP algorithm, with its ability to run concurrently across many nodes, further enhances the scalability of LDPC decoders [8,9].

Reinforcement-learning-based scheduling (RELDEC) has shown that machine learning can adaptively prioritize check node processing to improve decoding performance [2]. Its reported gains in error-correction performance validate the potential of advanced scheduling strategies. Yet RELDEC, in its current form, primarily targets scheduling update sequences for moderate-length codes, which can limit its effectiveness for large-scale parallel processing of latency-critical medium-to-long LDPC codes. Meanwhile, a high-throughput LDPC decoder introduced in [15] illustrates how pipeline reuse and careful memory-conflict mitigation can achieve 100 Gb/s throughput. The combination of learning-guided scheduling with hardware-friendly pipeline parallelism pushes LDPC decoding further, reducing iteration latency for large, latency-critical LDPC codes while retaining robust performance.

In contrast to sequential RL-based decoding frameworks such as RELDEC, our goal is to adapt reinforcement learning to parallel architectures by forming clusters of check nodes that are guaranteed to be conflict-free during row-parallel updates.

RELDEC focuses on sequential scheduling, where its RL model is optimized for improved error-correction performance by forming clusters that maximize within-cluster dependencies. In our work, we adopt the RL training formulation and RL environment definition from RELDEC, but extend the method toward parallel architectures. Here, clusters must satisfy two-edge independence, ensuring that their CNs can be updated simultaneously without memory conflicts. Our framework applies reinforcement learning to row-parallel decoding and emphasizes latency reduction and conflict mitigation rather than sequential error rate optimization.

Our contributions are as follows:

- We propose a parallel LDPC decoding framework that adapts RL-based scheduling to row-parallel decoding, achieving high throughput with competitive error-correction performance.
- We introduce clustering strategies combined with learning-guided scheduling to reduce latency and mitigate memory conflicts in parallel decoding.
- We demonstrate that reinforcement learning can be integrated with hardware-friendly pipeline designs to explore the trade-off between throughput and error-correction performance in parallel LDPC decoding.

The rest of this paper is organized as follows. Section 2 presents preliminary concepts, including the fundamentals of coding theory, LDPC codes, and the background on RL and RELDEC, as well as the RL-based approach for LDPC decoding. Section 3 introduces the proposed clustering methodology for parallelization, describing the lifting-based, adaptive greedy heuristic, and On-the-Fly clustering methods. Section 4 details the proposed scheduling approaches and compares the computational complexity of the RELDEC and Q-Sum algorithms. The results are presented in Section 5, followed by a discussion in Section 6.

## 2. Preliminaries

In this paper, we adopt the following notation. Scalars are denoted by italic letters (e.g., *x*); vector quantities by bold lowercase letters (e.g., x); and matrices by bold uppercase letters (e.g., X). We denote [x]≜{0,…,x−1}, where *x* is a positive integer.

### 2.1. Coding Theory and Low-Density Parity-Check Codes

In coding theory, a binary linear code of length *n* and dimension *k* is defined as a *k*-dimensional subspace of $F_{2}^{n}$ and is denoted as an [n,k] binary linear code. Such a code can be characterized as the set of all solutions of Hx=0, where $H\in F_{2}^{m\times n}$ is a parity-check matrix with m≥n−k. The rate of the code is $R=\frac{n-\mathrm{rank}(H)}{n}=\frac{k}{n}$.

Linear codes can be represented via bipartite graphs, known as Tanner graphs [16]. The Tanner graph $G_{H}=\left(V\cup C,E\right)$ provides a graphical representation of a linear code defined by its parity-check matrix H. In this representation, the set of variable nodes is $V=\{v_{0},v_{1},\ldots ,v_{n-1}\}$ (corresponding to the columns of H), and the set of check nodes is $C=\{c_{0},c_{1},\ldots ,c_{m-1}\}$ (corresponding to its rows). An edge exists between a variable node $v_{i}$ and a check node $c_{j}$ if and only if $H_{ji}=1$. This graph not only visualizes the dependencies between code symbols and parity-check equations but also serves as the foundation for iterative, message-passing decoding techniques.

Binary linear codes with sparse parity-check matrices, in which the number of ones is much smaller than the total number of entries, are known as LDPC codes. The sparsity leads to Tanner graphs with a low average degree, a property that is crucial for reducing the computational complexity of iterative decoding. Originally introduced by Gallager in 1962 [17] and later adopted in modern standards such as 5G New Radio (5G-NR) [18], LDPC codes are widely recognized for their strong error-correction capability and their suitability for high-speed hardware implementation.

BP decoding is a widely used iterative message-passing algorithm for LDPC codes that operates on the Tanner graph representation [19]. It refines soft decisions based on the Tanner graph. Initially, each variable node is assigned an intrinsic log–likelihood ratio (LLR) derived from the channel observation $y_{v}$:

(1) $$L_{v}=\log\left(\frac{P(y_{v}|x_{v}=0)}{P(y_{v}|x_{v}=1)}\right)$$

At iteration *ℓ*, the message from a variable node *v* to a neighboring check node *c* is computed as

(2) $$m_{v\to c}^{\left(\ell \right)}=L_{v}+\sum_{c^{\prime }\in N\left(v\right)\setminus \left\{c\right\}}m_{c^{\prime }\to v}^{\left(\ell \right)}$$

where N(v) denotes the set of check nodes connected to *v*. In parallel, each check node updates its outgoing message to a variable node *v* according to

(3) $$m_{c\to v}^{\left(\ell \right)}=2\;\mathrm{atanh}\;\left(\prod_{v^{\prime }\in N\left(c\right)\setminus \left\{v\right\}}\tanh\;\left(\frac{m_{v^{\prime }\to c}^{\left(\ell -1\right)}}{2}\right)\right)$$

Following the computation of the messages $m_{v\to c}^{\left(\ell \right)}$ and $m_{c\to v}^{\left(\ell \right)}$ at iteration *ℓ*, the overall belief ${\hat{L}}_{v}^{\left(\ell \right)}$ for each variable node is updated as

(4) $${\hat{L}}_{v}^{\left(\ell \right)}=L_{v}+\sum_{c\in N(v)}m_{c\to v}^{\left(\ell \right)}$$

and a hard decision is made by setting

(5) $${\hat{x}}_{v}=\left\{\begin{array}{cc} 0, & {\hat{L}}_{v}^{\left(\ell \right)}\geq 0\\ 1, & {\hat{L}}_{v}^{\left(\ell \right)}<0 \end{array}\right.$$

The iterative process continues until a valid codeword is obtained (i.e., when $H{\hat{x}}^{⊤}=0$) or until a predetermined maximum number of iterations is reached. The inherent sparsity of H enables BP decoding to closely approximate maximum-likelihood decoding with a computational complexity that is well-suited for high-speed, hardware-based implementations [17,19].

### 2.2. Lifting and Array-Based QC-LDPC Codes

The lifting technique is used to construct large, structured parity-check matrices. In this process, a small base matrix is expanded by replacing each nonzero element with an arbitrary power of the circulant permutation matrix of size Z×Z, and each zero element with a Z×Z all-zero matrix [20]. Here, *Z* represents the lifting factor, which determines the size of these circulant blocks. This expansion produces a quasi-cyclic (QC) structure, which is a special type of LDPC code characterized by a parity-check matrix composed of blocks that are cyclic shifts of the identity matrix or all-zero blocks. QC structures are desirable because they simplify hardware implementation and decoding processes due to their inherent regularity and sparsity [11]. We refer to the layers of the lifted parity-check matrix as its block rows corresponding to single rows of the base matrix. When the base matrix is lifted by a factor *Z*, each layer corresponds to *Z* rows in the full parity-check matrix derived from one row of the base matrix.

For QC-LDPC codes, it is convenient to specify the lifted matrix using an exponent matrix. Let $S\in {\left(\left\{-1\right\}\cup \left[Z\right]\right)}^{m_{b}\times n_{b}}$ denote an exponent matrix, where $S_{i,j}=-1$ denotes a Z×Z all-zero block and $S_{i,j}=s\in \left[Z\right]$ denotes a Z×Z circulant permutation block with shift *s*.

$$H={\left[H_{i,j}\right]}_{i\in \left[m_{b}\right],\;j\in \left[n_{b}\right]},\;H_{i,j}=\left\{\begin{array}{cc} 0_{Z\times Z}, & S_{i,j}=-1,\\ P_{Z}^{\;S_{i,j}}, & S_{i,j}\in \left[Z\right], \end{array}\right.$$

Here $P_{Z}\in {\left\{0,1\right\}}^{Z\times Z}$ denotes the circulant permutation matrix that applies a one-position cyclic shift of the identity.

$$P_{Z}=\left(\begin{array}{ccccc} 0 & 0 & \ldots & 0 & 1\\ 1 & 0 & \ldots & 0 & 0\\ 0 & 1 & \ldots & 0 & 0\\ \vdots & \vdots & \ddots & \vdots & \vdots \\ 0 & 0 & \ldots & 1 & 0 \end{array}\right).$$

In this paper, we use an array-based LDPC (AB-LDPC) code, which is a structured subclass of QC-LDPC codes. We parameterize the array-based construction by (γ,p), where *p* is a prime number and γ<p. A standard block description of the corresponding array-based parity-check matrix $H_{\mathrm{AB}}\left(\gamma ,p\right)\in F_{2}^{\gamma p\times p^{2}}$ is

$$H_{\mathrm{AB}}\left(\gamma ,p\right)=\left(\begin{array}{ccccc} I_{p} & I_{p} & I_{p} & \ldots & I_{p}\\ I_{p} & P_{p} & P_{p}^{2} & \ldots & P_{p}^{p-1}\\ I_{p} & P_{p}^{2} & P_{p}^{4} & \ldots & P_{p}^{2(p-1)}\\ \vdots & \vdots & \vdots & \ddots & \vdots \\ I_{p} & P_{p}^{\gamma -1} & P_{p}^{2(\gamma -1)} & \ldots & P_{p}^{\left(\gamma -1)(p-1\right)} \end{array}\right),$$

where $I_{p}$ is the p×p identity matrix.

We obtain the QC code used in simulations by lifting the array-based parity-check matrix by factor *Z*. Equivalently, we set $m_{b}=\gamma p$ and $n_{b}=p^{2}$ and define an exponent matrix $S\in {\left(\left\{-1\right\}\cup \left[Z\right]\right)}^{\gamma p\times p^{2}}$ on the support of $H_{\mathrm{AB}}\left(\gamma ,p\right)$, with $S_{i,j}=-1$ when ${\left(H_{\mathrm{AB}}\right)}_{i,j}=0$ and $S_{i,j}\in \left[Z\right]$ when ${\left(H_{\mathrm{AB}}\right)}_{i,j}=1$. This produces a lifted parity-check matrix of size $\gamma pZ\times p^{2}Z$.

### 2.3. Reinforcement Learning

In the RL problem, an agent interacts with an environment that can be modeled as a finite Markov Decision Process (MDP) [21]. The agent selects actions that transition the environment between states and receives a reward associated with each action. The agent’s objective is to maximize the cumulative reward over a series of actions. This is achieved by using a policy that leverages an action-value function to estimate the effectiveness of an action in maximizing the expected long-term reward.

Suppose the environment allows *m* possible actions, and let the random variable $A_{\ell }\in \left[m\right]$, with realization *a*, represent the index of an action taken by the agent during learning step $\ell \in \{0,\ldots ,\ell_{\max}-1\}$. Let $S_{\ell }$, with realization $s^{\left(\ell \right)}\in S$, denote the current state of the environment before taking action $A_{\ell }$, and let $S_{\ell +1}$, with realization $s^{(\ell )\prime }$, represent the state of the MDP after executing $A_{\ell }$. The state space S contains all possible state realizations. Additionally, let $R_{\ell }=R\left(S_{\ell },A_{\ell },S_{\ell +1}\right)$ be the reward received at step *ℓ* for taking action $A_{\ell }$ in state $S_{\ell }$, resulting in state $S_{\ell +1}$.

Optimal policies for MDPs can be approximated using Monte Carlo methods such as Q-learning. The action-value function Q(S,A), also known as the Q-function, represents the expected long-term reward obtained by the agent after taking action $A_{\ell }$ in state $S_{\ell }$. During learning, the action-value function is iteratively updated for specific $\left(S_{\ell },A_{\ell }\right)$ pairs by incorporating the previously learned values and the reward $R_{\ell }$ obtained from the corresponding action. The optimal policy guides the agent to choose actions in a given state that maximize the Q-function value for that state. The Q-function can often be implemented as a Q-table, where each entry corresponds to a state-action pair’s estimated value.

### 2.4. RELDEC: Reinforcement Learning for LDPC Decoding

In RELDEC [2], sequential decoding of a moderate-length LDPC code is modeled as an MDP in which an RL agent optimizes the order of CN updates. Unlike conventional flooding-based BP, where all CNs and VNs are updated together, RELDEC considers clusters of CNs and schedules each cluster sequentially in every decoding iteration. These clusters are constructed to concentrate short cycles within a cluster, allowing the RL agent to exploit intra-cluster dependencies. This improves both convergence speed and overall bit error rate (BER) and frame error rate (FER) performance.

**Figure 1:**
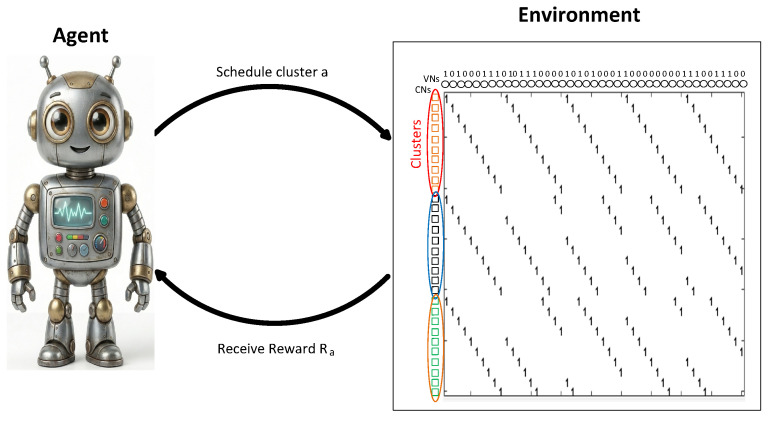
The state is defined by the hard-decision values of VNs within a cluster of CNs [2]. Circles denote variable nodes, squares denote check nodes, and colored circles indicate clusters. The right-hand side illustrates the corresponding parity-check matrix H, where filled entries represent ones and blank entries represent zeros.

RELDEC adopts the Q-learning algorithm, which iteratively refines a state-action value function during the learning stage. Here, an action corresponds to choosing a particular cluster of CNs. The state is defined by the hard-decision outputs of that cluster’s neighboring VNs; see Figure 1. After each update, the decoder’s newly computed posterior log–likelihood ratios (LLRs) for those VNs are mapped to 0/1 decisions, reducing the state space to a manageable size even for larger block-length codes. During training, the RL agent receives a reward reflecting the fraction of correctly decoded bits in the selected cluster. By continuously updating its Q-table based on these reward signals, the agent aims to learn an optimal CN cluster scheduling policy that improves decoding performance over many training examples.

Following the training, the decoding phase applies the learned policy by ranking all available clusters by their Q-values in the current decoding state at each iteration. Each cluster is sequentially scheduled based on the reinforcement learning-derived Q-values. The cluster with the highest Q-value is scheduled first, and then subsequent clusters are updated in descending order of Q-values. This procedure continues until either all parity checks are satisfied or a predefined iteration limit is reached. By prioritizing the most beneficial cluster updates, RELDEC achieves fewer unnecessary message passes and shows faster convergence than traditional flooding or other heuristic scheduling methods. Empirical results show that RELDEC offers notable gains in decoding efficiency and error-correction performance for a wide range of moderate-length LDPC codes.

In the special case where each cluster contains exactly one CN (cluster size of one), the method reduces to individually scheduling each CN. In such scenarios, the decoding order directly corresponds to prioritizing individual CN updates based on their specific Q-value rankings.

## 3. Clustering Methods for Parallel LDPC Decoding

### 3.1. Two-Edge Independent Clusters

We design our high-throughput decoding approach as follows. Clusters of check nodes are predefined before the decoding process begins, and the order of clusters in each decoder iteration is determined using Q-values learned via reinforcement learning. Each check node within a cluster is processed in parallel; therefore, check nodes in the same cluster must satisfy an independence condition to ensure a conflict-free decoding process. This independence is guaranteed by the property of two-edge independence, where check nodes do not share two-edge neighborhoods that could introduce dependencies during BP iterations (see Figure 1). In RELDEC, clusters are not required to satisfy two-edge independence; instead, they are constructed to maximize short cycles within a cluster.

**Definition** **1** (Two-edge Independence). *Let N(c) denote the set of all neighbors of a CN c, and let N(N(c)) represent the set of neighbors of these neighbors. Two check nodes $c_{1}$ and $c_{2}$ are said to be* two-edge independent *if:*

$$N\left(N\left(c_{1}\right)\right)\cap N\left(N\left(c_{2}\right)\right)=\emptyset .$$

**Figure 2:**
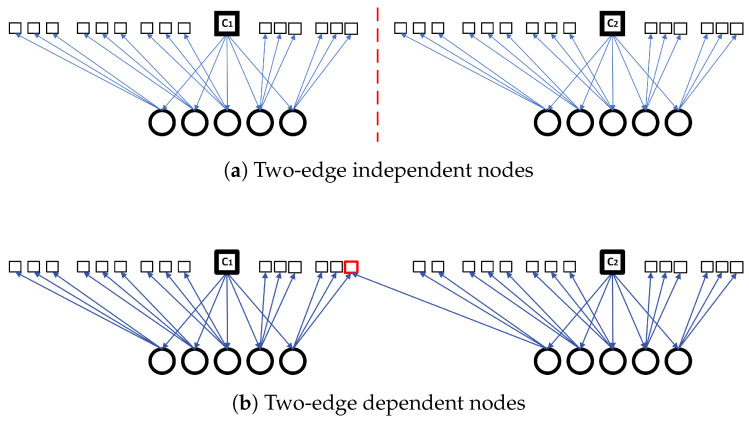
Graph representation illustrating two-edge independence. Circles denote variable nodes, squares denote check nodes, and solid edges represent graph connections.

Two-edge independence, shown in Figure 2, ensures that check nodes $c_{1}$ and $c_{2}$ can be updated concurrently without conflict within a single BP iteration. Since BP decoding involves message exchanges within a two-edge neighborhood, maintaining two-edge independence prevents interference between check nodes. This property thus enables efficient parallel processing within clusters and contributes to robust and reliable decoding performance.

Clusters are further used in our decoding algorithm (see Section 4). Below we describe three methods for forming two-edge independent clusters. The first two methods (Section 3.2 and Section 3.3) are offline and performed once, while the third method (Section 4.3) is dynamic and applied during the decoding run.

### 3.2. Clustering of Check Nodes: Lifting-Based Method

The structure of LDPC codes constructed via lifting allows clustering of check nodes for parallel processing. This clustering method is only applicable to LDPC codes constructed using lifting. For such codes, the two-edge independence required within clusters can be formally guaranteed, as stated in the following lemma.

**Lemma** **1.** *Let $H_{b}$ be a four-cycle-free base matrix and let H be its lifting by factor Z. Fix a base row i. The Z lifted check nodes (i,r), r∈[Z], are pairwise two-edge independent, i.e.,*

$$N\;\left(N(i,r_{1})\right)\cap N\;\left(N(i,r_{2})\right)=\emptyset \;\mathrm{for}\;r_{1}\neq r_{2}.$$

**Proof Sketch.**  Because each nonzero base entry corresponds to a power of a Z×Z circulant permutation block $P_{Z}$, each lifted check node (i,r) connects to exactly one VN in each nonzero column group [4,20]; hence $N\left(i,r_{1}\right)\cap N\left(i,r_{2}\right)=\emptyset$ for $r_{1}\neq r_{2}$.

Now suppose, toward a contradiction, that a check node $c^{\prime }$ belongs to both $N(N\left(i,r_{1}\right))$ and $N(N\left(i,r_{2}\right))$. Since $N\left(i,r_{1}\right)\cap N\left(i,r_{2}\right)=\emptyset$, the corresponding two paths must pass through two distinct column groups $j_{1}\neq j_{2}$. Then, in the base bipartite graph, there exists a row $i^{\prime }\neq i$ such that both rows *i* and $i^{\prime }$ are adjacent to both columns $j_{1}$ and $j_{2}$, forming a four-cycle in $H_{b}$, which contradicts the four-cycle-free assumption. Therefore the two-edge neighborhoods do not overlap.    □

**Figure 3:**
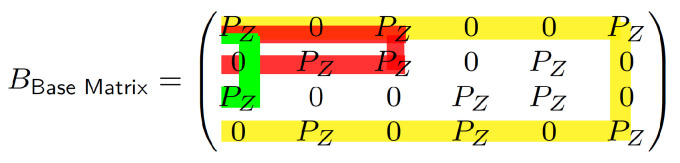
Illustration of lifting-based clustering in a base matrix. The color-coded groups highlight subsets of check nodes within the same layer that connect to distinct sets of variable nodes N(c) and N(N(c)).

Lemma 1 shows that the *Z* check nodes obtained by lifting a single base row (i.e., one layer) are pairwise two-edge independent. This guarantees that all check nodes in a layer can be updated concurrently within one BP iteration without conflicts, because their two-edge neighborhoods do not overlap. Figure 3 illustrates this point by showing that the dependencies of check nodes in a selected layer fall into other layers rather than within the same layer.

We form clusters by grouping check nodes within a layer, so two-edge independence holds by construction. The cluster size is a design parameter that sets the parallelism level and trades decoding latency against error-correction performance. In Section 5, we evaluate cluster sizes of 1, 2, 3, and 5. We refer to configurations where all clusters have the same cardinality as fixed-size clustering with cluster size *c*. Lifting-based clustering is deterministic and aligns with the quasi-cyclic structure of the lifted parity-check matrix, which simplifies hardware control and memory access compared with heuristic clustering methods. When $H_{b}$ contains four cycles, two-edge independence within a lifted layer is not guaranteed. In such cases, we use the proposed adaptive greedy heuristic clustering method in Section 3.3.

### 3.3. Clustering of Check Nodes: Adaptive Greedy Heuristic

Unlike the deterministic lifting-based clustering method, the adaptive greedy heuristic attempts to construct clusters by iteratively selecting check nodes that satisfy the two-edge independence criterion. This approach incrementally builds each cluster by consulting a check node adjacency table and validating independence constraints at every step. In contrast to lifting, which relies on structured properties of the code, the greedy method provides a flexible, general-purpose strategy applicable to any LDPC code.

The adaptive greedy heuristic clustering method operates as follows. A check node *c* is randomly chosen from the set of unclustered check nodes, and its inclusion in a cluster is validated against the two-edge independence condition. If the selected check node satisfies this constraint, it is added to the currently forming cluster. Otherwise, a failure counter is incremented. Once the failure count exceeds a predefined threshold, the algorithm removes a fraction of existing clusters and restarts to explore alternative configurations.

The cluster size *c* is a design parameter chosen according to hardware capabilities and resource constraints. Increasing *c* typically reduces decoding latency due to enhanced parallelization; however, our simulations (see Section 5) demonstrate a corresponding trade-off, as larger cluster sizes can negatively affect error-correction performance. Thus, practical values of *c* should be determined through experimental evaluation to balance throughput, latency, and decoding reliability.

Algorithm 1 summarizes the adaptive greedy heuristic clustering process. The algorithm starts by initializing the cluster set and relevant parameters (lines 1–3). At each iteration, a check node is randomly selected from the set of unclustered nodes and tentatively added to the current cluster (lines 5–6). If the two-edge independence criterion is satisfied, the node is kept in the cluster; once the cluster reaches the target size *c*, it is finalized and stored in the cluster set (lines 7–10). Otherwise, the node is removed and the failure counter is incremented (lines 12–14). When the number of failed attempts exceeds the predefined limit, a small portion of previously formed clusters is released back to the unclustered pool to expand the search space (lines 15–19). After all check nodes are processed, any remaining partial cluster is added to the set of clusters (lines 21–23), and the algorithm outputs the final clustering result (line 24).
**Algorithm 1** Adaptive Greedy Heuristic Clustering Method  1:**Input:** Parity-check matrix *H*, desired cluster size *c*, failure threshold limit.  2:**Output:** Set of clusters C satisfying two-edge independence.  3:Initialize C←∅, current←∅, fail←0.  4:**while** not all check nodes are clustered **do**  5:    Randomly select a check node c∉C∪current.  6:    Tentatively add *c* to current.  7:    **if** current satisfies two-edge independence **then**  8:        **if** |current|=c **then**  9:            Add current to C.10:            Reset current←∅.11:        **end if**12:    **else**13:        Remove *c* from current.14:        Increment fail←fail+1.15:    **end if**16:    **if** fail>limit **then**17:        Remove 20% of clusters from C.18:        Reset fail←0.19:    **end if**20:**end while**21:**if **current≠∅** then**22:    Add current to C as final incomplete cluster.23:**end if**24:**return** C.

The adaptive greedy heuristic is flexible and operates on a wide range of LDPC code structures without requiring predefined lifting constraints, and it iteratively forms clusters while ensuring the two-edge independence condition. However, its heuristic nature introduces non-determinism; different runs may yield different cluster configurations. Moreover, while the approach efficiently explores feasible clusterings, it does not guarantee the largest possible cluster sizes, potentially limiting the achievable level of parallelism. Despite these limitations, the adaptive greedy heuristic remains a useful alternative when structural lifting is unavailable or when greater flexibility in cluster formation is desired.

These clustering methods are offline phases of our proposed decoding framework. The full decoding algorithm, integrating clustering with RL-based scheduling, is presented in the next section.

## 4. RL-Based Scheduling for Parallel LDPC Decoding

Building on the clustering methods of Section 3, we now integrate clustering and scheduling into a unified decoding framework. Our proposed method consists of an offline setup phase, in which clusters and Q-values are computed, and an online decoding phase, in which clustered belief propagation is performed based on these precomputed quantities, as summarized in Algorithm 2.

**Figure 4:**
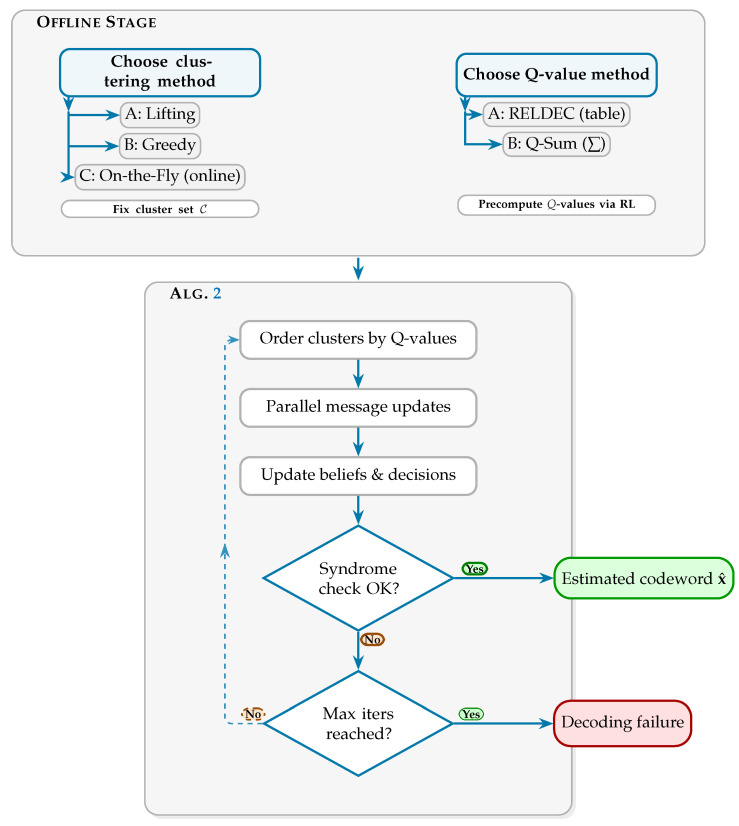
Decoding flow of the proposed LDPC decoding framework.

The overall decoding framework is illustrated in Figure 4, which includes both the offline and online stages. The offline stage defines the clustering strategy (Lifting or Greedy) and the Q-value computation method (RELDEC or Q-Sum). For On-the-Fly clustering, only per-check-node Q-values are computed offline. The online decoding stage is detailed in Algorithm 2, which performs clustered LDPC decoding based on the available cluster structure and Q-values.

In Algorithm 2, the decoder first computes the intrinsic LLRs from the received codeword (line 3). For each decoding iteration (line 4), clusters are sorted in descending order of their Q-values (line 5), and clusters are processed sequentially in that order (line 6). Within every cluster, check-to-variable and variable-to-check message updates are executed in parallel using the belief-propagation equations (lines 7–8, corresponding to Equations (3) and (2)). After message updates, posterior beliefs are refined (line 9), and hard decisions are made for all variable nodes (line 10). The decoder then verifies the syndrome condition $H{\hat{x}}^{⊤}=0$ (line 12); if satisfied, decoding terminates successfully (line 13). Otherwise, the algorithm proceeds to the next iteration (line 15) until the maximum iteration count is reached. Finally, the decoded codeword is returned as the output (line 16).
**Algorithm 2** Clustered LDPC Decoding with Predefined Clusters  1:**Input:** *H*, received codeword *y*, precomputed cluster set C, Q-values $Q(C_{k})$, maximum decoding iterations $\ell_{\max}$  2:**Output:** Estimated codeword $\hat{x}$  3:Compute intrinsic LLRs $L_{v}$ from received codeword *y* using Equation (1).  4:**for** each decoding iteration $\ell =1,2,\ldots ,\ell_{\max}$ **do**  5:    Sort clusters in C by descending order of Q-values $Q(C_{k})$.  6:    **for** each cluster $C_{k}\in C$ (in sorted order) **do**  7:        In parallel, for each check node $c\in C_{k}$, compute check-to-variable messages $m_{c\to v}^{\left(\ell \right)}$ (Equation (3)).  8:        In parallel, for each connected variable node *v*, compute variable-to-check messages $m_{v\to c}^{\left(\ell \right)}$ (Equation (2)).  9:    **end for**10:    Update posterior beliefs ${\hat{L}}_{v}^{\left(\ell \right)}$ (Equation (4)).11:    Make hard decisions ${\hat{x}}_{v}$ (Equation (5)).12:    **if** $H{\hat{x}}^{⊤}=0$ **then**13:        **break** (successfully decoded)14:    **end if**15:**end for**16:**return** estimated codeword $\hat{x}$.

In sequential LDPC decoding, scheduling refers to determining the optimal order in which CNs are processed to maximize convergence speed and decoding performance. Various scheduling strategies have been proposed in literature, each aiming to improve error-correction performance and convergence efficiency. Examples include informed dynamic scheduling, which prioritizes nodes based on reliability metrics [7,10], layered decoding methods known for improving throughput [11], and GPU-based parallel scheduling techniques for high-speed decoding [8,14].

In this work, we focus specifically on two distinct approaches: the RELDEC-based method and the Q-Sum method. Both methods utilize Q-values, derived via reinforcement learning techniques as introduced by Habib et al. [2,22], to determine the optimal sequence of CN updates. However, the methods differ significantly in how cluster Q-values are computed and stored. The RELDEC-based method computes Q-values explicitly for entire clusters of check nodes, leading to exponentially increasing storage requirements as cluster size grows. In contrast, the Q-Sum method approximates cluster-level Q-values by summing the individual Q-values of the check nodes within a cluster, greatly reducing storage complexity from exponential to linear. These differences directly impact memory usage, computational complexity, and practical scalability of the decoding process.

### 4.1. Cluster Scheduling via RELDEC

As explained in Section 2.4, the RELDEC method formulates LDPC decoding scheduling as an MDP, employing an RL agent to assign Q-values to clusters of check nodes based on their impact on decoding performance. In this paper, we directly utilize the RELDEC-derived Q-values, learned offline, to schedule check node clusters, prioritizing updates according to these learned values during each decoding iteration.

While effective in enhancing decoding performance, RELDEC’s approach of explicitly computing and storing Q-values for entire clusters results in an exponentially large Q-table with respect to the cluster size. Specifically, Habib et al. demonstrated that RELDEC maintains a Q-table of size $\max_{a}\left(2^{l_{a}}\right)\times \left\lceil \frac{m}{c}\right\rceil$, where $l_{a}$ is the number of variable node neighbors connected to each cluster, *m* is the total number of CNs, and *c* is the cluster size [2]. This exponential growth imposes significant memory and computational complexity, creating scalability challenges for high-throughput and large-scale decoding architectures. These limitations directly motivate the development of our proposed Q-Sum method, described in the next subsection, which substantially reduces memory requirements and computational overhead.

### 4.2. Q-Sum Method

To mitigate the scalability issue of RELDEC, we propose the Q-Sum method, which approximates cluster-level Q-values by summing the individual Q-values of each check node in the cluster. Instead of maintaining an exponentially growing table, Q-Sum derives a cluster’s priority from the sum of the individual Q-values of the check nodes within the cluster. Formally, given a cluster *C* containing check nodes $c_{1},c_{2},\ldots ,c_{c}$, its scheduling priority is computed as:

(6) $$Q_{\mathrm{sum}}\left(C\right)=\sum_{i=1}^{c}Q\left(c_{i}\right),$$

where $Q(c_{i})$ represents the Q-value of an individual check node, learned offline using the RELDEC method with cluster size 1. This significantly reduces memory requirements, as only O(m) values need to be stored, where *m* is the total number of check nodes in the parity-check matrix. This approximation neglects higher-order interactions between check nodes within a cluster, trading some scheduling optimality for substantially reduced storage and computational complexity.

The Q-value of a check node intuitively approximates how beneficial it is to schedule that check node earlier within the same decoding iteration rather than later. Summing the Q-values of the check nodes within a cluster therefore provides an approximation of how beneficial it is, from a decoding perspective, to schedule an entire cluster of check nodes within one iteration. This approximation also explains a potential loss in error-correction performance: a check node that would ideally be scheduled much later in an iteration may be grouped into the same cluster as check nodes that are better scheduled earlier. As a result, the entire cluster may be forced to be scheduled at a non-optimal position in the iteration. This effect can degrade error-correction performance, while simultaneously enabling high throughput by allowing parallel scheduling of all check nodes within the cluster. Therefore, Q-Sum reflects an inherent trade-off: sacrificing a small amount of error-correction performance relative to state-of-the-art sequential decoding algorithms in order to achieve higher throughput through parallel check-node decoding. In two special cases Q-Sum becomes optimal: when all Q-values of the check nodes in a cluster are either among the highest or among the lowest compared to all other check nodes.

The Q-Sum method retains the core principles of RL-based scheduling while ensuring feasibility for large-scale LDPC decoding. By approximating cluster Q-values through summation, it enables efficient decoding order selection without the need for an exponentially large Q-table. This trade-off between precision and computational efficiency makes Q-Sum particularly attractive for practical implementations.

### 4.3. On-the-Fly Clustering

Unlike lifting-based and adaptive greedy heuristic clustering methods, which rely on clusters constructed in advance, the On-the-Fly Clustering method dynamically determines the decoding order of check nodes during the decoding process. Instead of statically assigning check nodes to clusters, this method processes check nodes sequentially while ensuring that consecutive nodes maintain the required two-edge independence.

The decoding order in the On-the-Fly Clustering method is determined by a scheduling mechanism based on the Q-values of individual CNs computed in the offline phase (see Algorithm 2). Specifically, this method can be viewed as a special case of clustered decoding, where the effective cluster size is one. To ensure the two-edge independence constraints dynamically during decoding, an adjacency lookup table of size m×(m−1) is maintained. This table explicitly encodes two-edge dependency relationships among check nodes, identifying nodes that can be processed without causing conflicts.

During decoding, the scheduling order provided by the Q-values (Algorithm 2, line 5) determines the processing order, while the per-cluster loop (line 6) is interpreted with c=1 so that check nodes are scheduled individually. Each time a check node is selected, the adjacency lookup table is consulted to verify its two-edge independence with previously processed nodes. If a dependency is detected, the processing of the dependent check node is deferred until all conflicting nodes have been processed.

The On-the-Fly Clustering method offers several advantages. Since clustering decisions are made dynamically, it eliminates the need for predefined clustering structures, making it highly adaptable to varying LDPC code configurations. It also enables optimized cluster ordering during decoding, since check nodes can be scheduled flexibly and individually without the constraints imposed by predefined clustering.

Such flexibility allows the scheduling sequence to more closely follow the ordering induced by the Q-values, improving convergence behavior while maintaining error-correction performance comparable to RELDEC.

Indeed, RELDEC demonstrated that using a cluster size of one can yield strong error-correction performance compared to larger cluster sizes, albeit without parallel processing. However, this flexibility comes at the cost of increased computational overhead due to frequent dependency checks. Additionally, it requires extra cache memory to store and efficiently access the adjacency lookup table. These trade-offs become especially significant when deploying the On-the-Fly Clustering method in systems where a high degree of parallelism is required, as the size of the adjacency lookup table grows rapidly with increased parallel processing demands.

The On-the-Fly Clustering method can be implemented by adapting Algorithm 2 with minimal modifications. Specifically, replace the per-cluster loop (line 6) with a per-check-node loop that enforces two-edge independence before executing the message updates (lines 7–8). Keep the Q-value ordering step (line 5) but apply it to individual check nodes, maintaining an effective cluster size of c=1. For each scheduled check node, the adjacency lookup table is consulted to ensure two-edge independence from previously processed nodes. If a check node violates this condition, its processing is deferred until the independence requirement is satisfied. This modification ensures that independence is dynamically maintained without predefined clustering constraints.

### 4.4. Comparison of the Methods

The fundamental difference between RELDEC and Q-Sum lies in how Q-values of clusters are computed and stored. While RELDEC maintains an exhaustive Q-table to track all possible states of a cluster, Q-Sum adopts a linear approximation that scales efficiently with an increasing number of check nodes. Table 1 summarizes the key distinctions between our approaches.

The RELDEC method stores a Q-table that is exponential in cluster size, whereas Q-Sum stores only O(m) per-CN Q-values. On-the-Fly additionally stores a boolean adjacency lookup table encoding two-edge dependencies, which requires $O(m^{2})$ memory. For scheduling, all methods need to sort clusters by their Q-values in each iteration.

## 5. Simulation Results

This section presents the performance evaluation of the proposed clustering, scheduling, and parallel processing methods. We compare the BER, FER, and decoding latency of the proposed RELDEC method, Q-Sum method, and On-the-Fly clustering against conventional approaches such as flooding-based BP and fixed-order scheduling.

**Figure 5:**
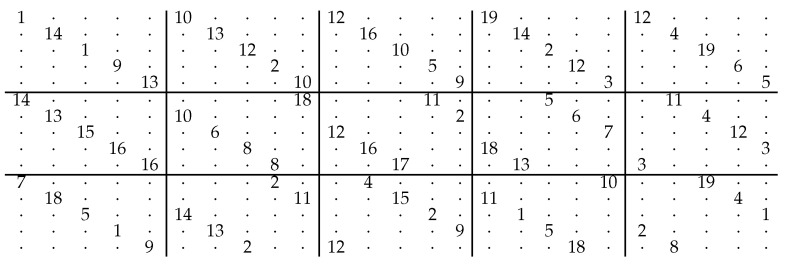
Base matrix of the lifted array-based LDPC code used in our simulations. Dots (·) represent Z×Z all-zero submatrices, while each integer indicates the left cyclic shift of a Z×Z identity matrix, corresponding to a circulant permutation. The structure reflects a (γ=3,p=5) array-based code with lifting size Z=20, resulting in a block length of 500 bits.

Evaluations are conducted using two codes. The first one is a lifted AB-LDPC code [20], characterized by the parity-check matrix H(γ,p) with parameters γ=3, p=5, and lifting size Z=20, resulting in a codeword length of 500 bits. The corresponding base matrix is illustrated in Figure 5, where dots represent z×z zero matrices, and integers represent cyclically shifted identity matrices. Each integer specifies the number of leftward cyclic shifts applied to a z×z identity matrix. This array-based LDPC code is representative of structured LDPC codes commonly used in scheduling and parallel decoding studies, and its regular structure facilitates controlled evaluation of clustering and scheduling effects. All simulations were performed using the all-zero codeword. A fixed maximum of 50 iterations was adopted as the decoding termination criterion, which is a commonly used setting in LDPC decoding studies.

**Figure 6:**
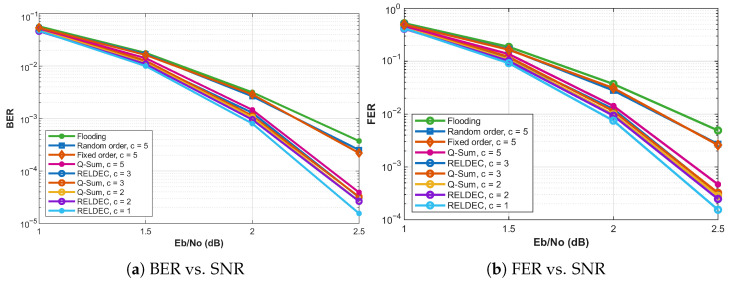
Comparison of BER and FER across different scheduling approaches for the array-based LDPC code described in Figure 5.

In all experiments with cluster size *c* (see Figure 6), the clusters are fixed in advance and are identical for all methods that use static clustering. For a given *c*, the curves labeled “RELDEC, c=k” and “Q-Sum, c=k” therefore use the same fixed-size clusters and differ only in how cluster priorities are computed. In the RELDEC scheme, Q-values are learned directly for clusters of size *k* using a cluster-level Q-table, which results in exponential storage growth with *k*. In the Q-Sum scheme, cluster Q-values are obtained by summing per-check-node Q-values learned once with cluster size one, as in (6), which reduces storage to linear in the number of check nodes. The “Random order, c=5” and “Fixed order, c=5” baselines also operate on the same fixed-size clusters but schedule clusters without reinforcement learning. The “On-the-Fly” scheme does not use precomputed clusters and instead enforces two-edge independence dynamically at run time using per-check-node Q-values.

All evaluations are performed over an AWGN channel. The On-the-Fly Clustering method demonstrates robust performance, particularly at higher SNRs, achieving error-correction performance comparable to RELDEC. While it exhibits slightly higher BER relative to RELDEC, this minor degradation is offset by significantly reduced memory usage and the elimination of large cluster-level Q-tables, at the cost of additional online clustering overhead. This outcome underscores the advantage of adaptive scheduling strategies during dynamic cluster construction, effectively balancing decoding accuracy and complexity. Furthermore, the On-the-Fly method achieves reduced decoding latency compared to RELDEC, making it well-suited for high-throughput decoding scenarios. The Q-Sum method similarly achieves a favorable trade-off between latency and decoding accuracy, providing a scalable alternative to RELDEC.

**Figure 7:**
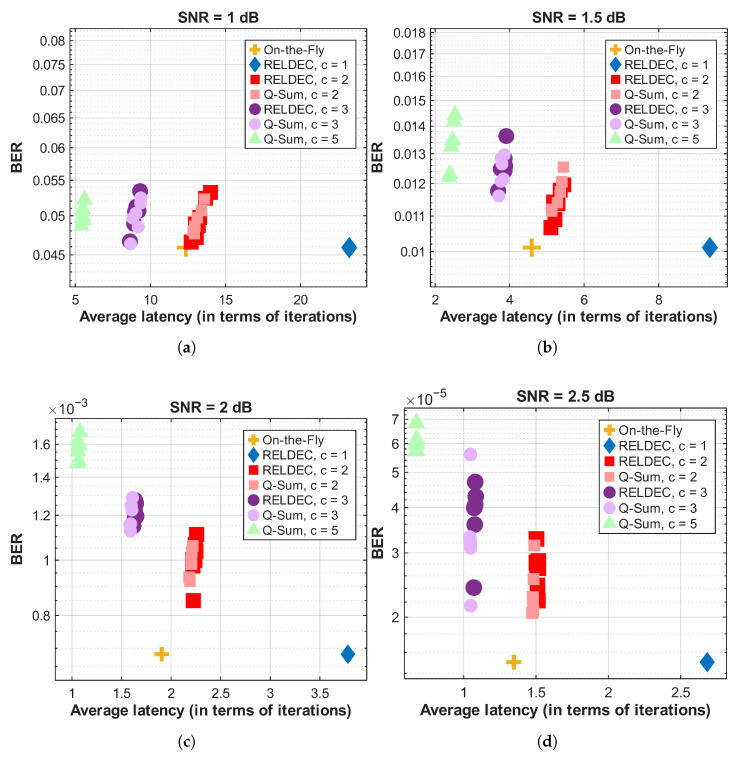
BER versus average latency under different SNRs. (**a**) SNR = 1 dB. (**b**) SNR = 1.5 dB. (**c**) SNR = 2 dB. (**d**) SNR = 2.5 dB. Average latency is measured in decoding iterations.

Decoding latency plays a crucial role in high-throughput applications and is evaluated using the number of decoding iterations required for successful decoding. We adopt a normalized latency model in which one iteration of fully sequential decoding corresponds to one unit of time, and parallel processing of check nodes within each cluster reduces the effective iteration time proportionally to the cluster size. Figure 7 presents the trade-off between BER and average latency for different scheduling approaches at SNR levels of 1 dB, 1.5 dB, 2 dB, and 2.5 dB. The results reveal that larger cluster sizes lead to lower decoding latency since more check nodes are processed in parallel within each iteration. However, this reduction in latency comes at the cost of slightly degraded error-correction performance, as seen in Q-Sum clustering with large cluster sizes.

As shown in Figure 7, increasing the cluster size results in a lower number of required iterations, thus reducing decoding latency. However, this comes at the expense of a slight degradation in BER. The RELDEC method achieves the best error-correction performance, but its computational complexity scales exponentially with cluster size, making it impractical for large-scale applications. The Q-Sum method offers a more scalable alternative by approximating cluster Q-values through summation rather than maintaining an exponentially large Q-table. This approximation allows for efficient scheduling while maintaining strong decoding performance.

The experimental evaluation highlights several important observations. First, larger clusters enable lower latency by allowing more check nodes to be processed in parallel. However, this benefit comes with a slight increase in BER, particularly when using heuristic scheduling strategies. The RELDEC method provides the best error-correction performance but is constrained by its limited latency and exponential memory growth. The Q-Sum method mitigates these limitations by using a summation-based approximation for cluster Q-values, making it an alternative for large-scale LDPC decoding. Finally, the On-the-Fly Clustering method demonstrates an advantage in terms of flexibility and computational efficiency, offering an effective scheduling approach that balances decoding accuracy and latency. However, its performance advantage comes with increased overhead due to frequent dependency checks, as well as additional memory requirements for storing and accessing the adjacency lookup table, which should be considered in practical high-throughput implementations.

At the offline learning stage, the Q-table was trained iteratively at SNR values of 1, 1.5, 2, and 2.5 dB. Once obtained, this Q-table was fixed and reused for decoding across all SNR levels.

In Figure 7, each point corresponds to an independent decoding simulation at the indicated SNR value. For a fixed cluster size, RELDEC and Q-Sum exhibit closely matched error-correction performance. Each point corresponds to an independent decoding simulation at the indicated SNR value. In particular, the BER points for cluster sizes c=2 (pink and red) and c=3 (light and dark purple) closely overlap across the considered SNR range. This indicates that, when the degree of parallelism is held constant, the Q-Sum approximation does not introduce a noticeable additional BER penalty beyond that imposed by clustering itself. As expected, RELDEC with smaller cluster sizes or sequential scheduling achieves the best overall error-correction performance.

The results also show that increasing the cluster size reduces decoding latency at the cost of a slight degradation in error-correction performance, most visible around an SNR of 2 dB. At higher SNR values, such as 2.5 dB, Q-Sum effectively balances decoding accuracy and latency, making it a scalable alternative to RELDEC. Overall, RELDEC offers the strongest error-correction performance but faces scalability limitations, whereas Q-Sum and On-the-Fly Clustering achieve lower latency with manageable computational complexity.

The second code we consider is a 5G-NR LDPC code based on BG2 with block length 520 and information length 100 bits. Since its base matrix contains four cycles, the lifting-based clustering method is not applicable. For this reason, we employ the On-the-Fly clustering method.

We also evaluated the adaptive greedy heuristic clustering method from Section 3.3 on the 5G-NR BG2 code; however, due to the dense two-edge dependency structure, it failed to form valid clusters even for size c=2, and therefore no greedy-based parallel results are reported.

**Figure 8:**
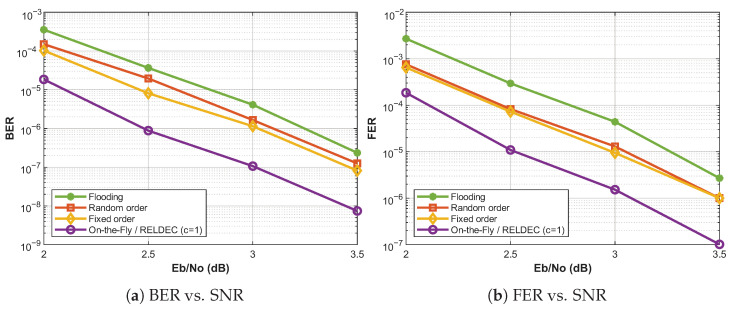
BER and FER results for a [520, 100] 5G-NR LDPC code using multiple BP decoding methods over an AWGN channel with $I_{\max}=50$.

Figure 8 shows the BER and FER performance of a 5G-NR LDPC code under four decoding schedules: flooding, fixed-order sequential, random-order sequential, and the proposed On-the-Fly method. The On-the-Fly curve corresponds to scheduling based on per-check-node Q-values learned using the RELDEC framework with cluster size c=1, while two-edge independence is enforced dynamically using the adjacency lookup table, as described in Section 4.3, to allow parallel updates. The observed behavior only affects latency rather than error-correction performance.

Table 2 shows only a marginal latency reduction for the 5G-NR BG2 code. This limited improvement is attributed to the irregular structure of the parity-check matrix, which restricts exploitable parallelism under the On-the-Fly independence constraint. For the considered code, the corresponding adjacency lookup table has a density of about 0.011, indicating that, on average, only about 1.1% pairs of check nodes can be scheduled in parallel. As a result, dynamic parallel scheduling yields modest iteration savings despite preserving error-correction performance.

By contrast, the AB-LDPC code considered earlier has an adjacency lookup table density of 0.74, indicating that a large fraction of check-node pairs satisfy the two-edge independence condition. This difference in adjacency density is reflected in the relative latency reductions observed for the two codes under On-the-Fly scheduling.

## 6. Discussion

In this study, we introduced an RL-based decoding approach combined with pre-clustered scheduling strategies to address the inherent trade-off between decoding throughput and error-correction performance in LDPC decoding. Our results demonstrate significant throughput and efficiency improvements compared to conventional decoding methods. Baseline flooding decoding methods, despite their simplicity, typically show limitations in both latency and error-correction performance. Sequential decoding algorithms, while providing superior error-correction performance, compromise throughput because of limited parallel execution capabilities.

The proposed On-the-Fly Clustering method, built upon the two-edge independence criterion, effectively balances decoding efficiency and complexity. Simulation results show that this method achieves decoding performance comparable to the established RELDEC approach, but with substantially reduced computational complexity and latency. These findings align with recent studies highlighting that strategic clustering combined with optimized scheduling significantly improves decoding efficiency and reduces latency.

Additionally, the scalability of the proposed methods is primarily determined by their memory complexity. The Q-Sum scheduling method introduced in this work significantly reduces storage complexity by approximating cluster-level Q-values, effectively lowering the complexity from exponential to linear. The On-the-Fly clustering method requires an additional $O(m^{2})$ binary dependency lookup table, which becomes increasingly costly for long block lengths, whereas Q-Sum retains linear memory growth and is therefore more suitable for longer codes.

This simplification is particularly advantageous for scalable deployments and aligns well with the evolving requirements of modern wireless communication systems. Thus, the Q-Sum approach emerges as a viable and practical solution for large-scale implementations.

The ability of our framework to dynamically prioritize clusters based on RL-derived Q-values notably improves LDPC decoding in real-time scenarios. This is relevant for future low-latency communication, where dynamic channel conditions and latency constraints present substantial challenges. The method’s compatibility with a wide range of LDPC codes further enhances its value in practical communication scenarios.

This study targets the core trade-off in LDPC decoding: improving error-correction performance at high-throughput, low-latency operation. By combining RL-based sequential decoding with a variety of clustering methods for parallel decoding, we achieve reliable decoding at reduced latency and complexity.

## 7. Conclusions

We presented a reinforcement learning-based decoding framework that addresses the trade-off between error-correction performance and latency in LDPC decoding. By combining RL-guided scheduling with effective clustering strategies, including the On-the-Fly and Q-Sum methods, our approach enables scalable, high-throughput decoding with reduced complexity. The proposed techniques are well-suited for future low-latency communication systems, offering practical solutions for next-generation wireless standards.

## Figures and Tables

**Table 1:** Comparison of RELDEC, Q-Sum, and On-the-Fly scheduling methods.

Feature	RELDEC Method	Q-Sum Method	On-the-Fly Method
Q-value computation	Cluster-based	Sum of CN Q-values	Individual CN Q-values
Storage complexity	$O\;\left(2^{c}\frac{m}{c}\right)$	O(m)	$O(m^{2})$
Online scheduling overhead	$O\;\left(\frac{m}{c}\log\frac{m}{c}\right)$	$O\;\left(\frac{m}{c}\log\frac{m}{c}\right)$	$O\;\left(m\log m\right)$

**Table 2:** Average decoding latency (in iterations) for the 5G-NR BG2 code under On-the-Fly parallel scheduling and a sequential (no-parallel) baseline.

Method	$E_{b}/N_{0}$ = 2 dB	2.5 dB	3 dB	3.5 dB
On-the-Fly Parallel	2.439	2.175	2.051	1.993
RELDEC Sequential	2.487	2.218	2.091	2.033

## Data Availability

Data are contained within the article.
